# Efficacy of aluminum chloride in severe regorafenib-associated hand-foot skin reactions: a single-arm trial

**DOI:** 10.1186/s12885-023-10864-9

**Published:** 2023-05-04

**Authors:** Aya Nishizawa, Eiji Shinozaki, Takeru Wakatsuki, Takahiro Satoh, Naoya Yamazaki, Shunsuke Oyamada, Keisuke Ariyoshi, Kota Kihara, Masahiro Tsuboi, Kensei Yamaguchi

**Affiliations:** 1 Department of Dermatology, Cancer Institute Hospital, Japanese Foundation for Cancer Research, Koto-ku, Tokyo, Japan; 2 Department of Dermatology, Tokyo Metropolitan Cancer and Infectious Disease Center, Komagome Hospital, 3-18 Honkomagome, Bunkyo-ku, Tokyo, 113-8677 Japan; 3 Department of Gastroenterological Chemotherapy, Cancer Institute Hospital, Japanese Foundation for Cancer Research, Koto-ku, Tokyo, Japan; 4 Department of Dermatology, National Defense Medical College, Saitama, Japan; 5 Department of Dermatology, National Cancer Center Hospital, Chuo- ku, Tokyo, Japan; 6 Japanese Organization for Research and Treatment of Cancer (JORTC) Data Center, Tokyo, Japan; 7 Japanese Organization for Research and Treatment of Cancer (JORTC) Operations Office, Tokyo, Japan; 8 Department of Thoracic Surgery, National Cancer Center Hospital East, Chiba, Japan

**Keywords:** Hand-foot skin reactions, Multi kinase inhibitors, Regorafenib, Aluminum chloride

## Abstract

**Background:**

Regorafenib, a multikinase inhibitor, causes a high frequency of hand-foot skin reactions (HFSRs). The present study evaluated the efficacy of topical aluminum chloride, a perspiration suppressant, in reducing the severity of hand-foot skin reactions (HFSRs) caused by regorafenib.

**Methods:**

The present single-arm study included patients with metastatic colorectal cancer receiving regorafenib. Aluminum chloride ointment was applied topically one week prior to the start of regorafenib treatment, and the observation period was 12 weeks. The primary endpoint was the incidence of regorafenib-related grade 3 HFSR. Secondary endpoints were the incidence of all grades of HFSR, time to any grade of HFSR, time to improvement from grade 2 or higher to grade 1 or lower, treatment discontinuation rate, treatment interruption rate or dosage reduction due to HFSR, and incidence of adverse effects of aluminum chloride.

**Results:**

In total 28 patients were enrolled, and 27 patients were analyzed. The incidence of grade 3 HFSR was 7.4%, meeting the primary endpoint. The incidence of all grades of HFSR was 66.7%, and the median time to the occurrence of any grade of HFSR was 15 days. No patients discontinued or reduced the regorafenib dosage because of HFSR. The most common reason for the interruption of regorafenib therapy was liver dysfunction in nine patients (33%) and HFSR in three patients (11%). No serious adverse events related to aluminum chloride were observed.

**Conclusions:**

Aluminum chloride ointment, a drug commonly used in routine practice to treat hyperhidrosis, is safe to use, has no serious side effects, and may be effective in reducing the occurrence of severe, regorafenib-related HFSR.

**Trail registration:**

ClinicalTrials.gov. identifier: jRCTs031180096, Registered on 25/01/2019.

**Supplementary Information:**

The online version contains supplementary material available at 10.1186/s12885-023-10864-9.

## Background

In recent years, mortality and morbidity associated with colorectal cancer (CRC) have significantly increased in Japan [1]. Recent clinical trials have demonstrated that several anticancer agents are effective in improving the outcomes of metastatic CRC (mCRC). One of these is regorafenib, an oral multikinase inhibitor (MKI) that blocks the activity of multiple protein kinases involved in oncogenes (KIT, RET, RAF, and BEAF), tumor angiogenesis (VEGFR1, VEGFR2, VEGFR3, and TIE2), and the tumor microenvironment (PDGFR and FGFR). Regorafenib is indicated for the treatment of mCRC and has also been approved for the treatment of gastrointestinal stromal tumors and hepatocellular carcinoma (CORRECT, [2, 3] GRID, [4] and RESORCE trials, [5] respectively).

MKI treatment is associated with a variety of adverse events (AEs) that can significantly impact health-related quality of life (QOL). The most frequent of these AEs is hand-foot skin reaction (HFSR), which is characterized by the appearance of painful erythematous lesions localized to the palms and soles, typically early after MKI administration, followed by blistering and keratotic lesions. Prompt management of HFSR is required as symptoms often lead to treatment interruption, dosage reduction or discontinuation.

Regorafenib is associated with a high incidence of HFSR, with previous studies reporting an incidence of 47% for any grade and 17% for grade 3 HFSR in the CORRECT trial [2, 3]. HFSR appears to be more common among Japanese patients; the Japanese subpopulation in the CORRECT trial demonstrated an incidence of 80% for all grades of HFSR and 30% for grade 3 HFSR [3]. Severe HFSR frequently causes treatment interruption or dosage reduction. Indeed, more than 10% of the Japanese subpopulation in the CORRECT trial required treatment discontinuation. Although early clinical trials used an initial dosage of 160 mg, it has become common to start regorafenib at a lower dosage to decrease the risk of adverse effects. Some clinical trials have reported a 20% incidence of grade 3 HFSR at a starting dosage of 120 mg [6, 7].

HFSR typically develops within one to two weeks of MKI administration and may occur frequently within the first two months of treatment [8, 9]. Although the mechanisms underlying the pathogenesis of HFSR remain unclear, damage incurred by epidermal cells and eccrine sweat glands during drug excretion is thought to be involved [10, 11]. As PDGFR and c-KIT are expressed in eccrine sweat gland tissue, sweat gland abnormalities caused by the inhibition of PDGFR and c-KIT by MKI may also be a contributory factor [11]. Moreover, a study examining the relationship between MKI-related HFSR and sweating reported that sweat samples from the subjects contained drug metabolites [12, 13]. Therefore, drug metabolites contained in sweat may be implicated in the development of HFSR.

Currently, high-dose topical corticosteroids, urea-based topical creams (UBCs) that inhibit keratinization, [14] and dressings for pain control and skin protection are used for symptom control and preventing HFSR [15]. However, there is a clinical need for new treatment options as these methods are not very efficacious. A previous study reported that aluminum chloride was effective in inhibiting the development of liposomal doxorubicin-related HFSR [16]. Aluminum chloride ointment suppresses sweating by blocking the secretion of sweat by the skin and is used in general practice as a treatment for hyperhidrosis.

Therefore, based on the hypothesis that aluminum chloride might be useful in preventing regorafenib-related HFSR, the present study aimed to evaluate the efficacy of topical aluminum chloride in reducing HFSR severity in mCRC patients receiving regorafenib.

## Methods

### Study design

The present, single-arm, nonrandomized, observational study was conducted at the Cancer Institute Hospital of the Japanese Foundation for Cancer Research from January 2019 to January 2022. All the data were collected and de-identified using an electronic data capturing system (Viedoc™, Pharma Consulting Group, Ltd.). An independent data and safety monitoring committee monitored the safety and progress of the trial.

### Subjects

**Fig. 1:**
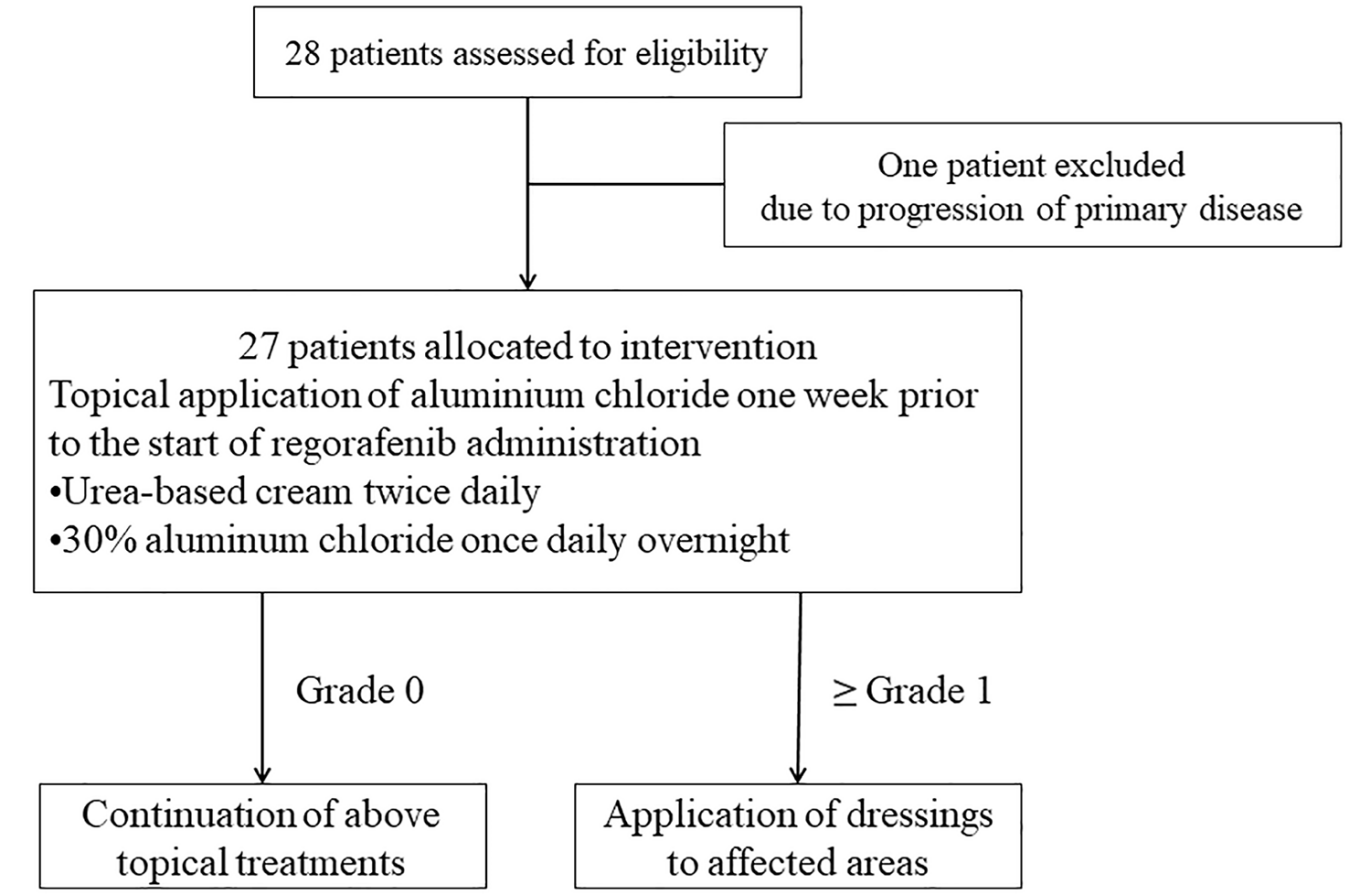
CONSORT diagram

Inpatients and outpatients being treated for mCRC at the Cancer Institute of Hospital Ariake who met the eligibility criteria without meeting any of the exclusion criteria were included. Topical aluminum chloride was applied one week prior to the start of regorafenib treatment. Regorafenib 120 mg, a lower starting dosage than normally prescribed in the study center’s routine clinical practice, was administered once daily for 21 days in each 28-day cycle. The inclusion and exclusion criteria are listed in eTable 1 [see Additional file 1]. Figure 1 shows the CONSORT diagram, and eTable 2 shows the treatment flow [see Additional file 1]. Dosage reduction or treatment interruption was allowed at any time according to the severity of the AEs caused by regorafenib or aluminum chloride (eTable 3 [see Additional file 1]). The treatment period was 12 weeks. Treatment was terminated in cases meeting the criteria for discontinuation (eTable 2 [see Additional file 1]).

The 30% aluminum chloride formulation used in the present study was dispensed in the hospital. The method of dispensing the drug is shown in eTable 4 [see Additional file 1]. If HFSR ≥ grade 1 developed, dressings were applied to the affected area (eFigure 1 [see Additional file 1]).

### Endpoints and definitions

The primary endpoint of the present, single-arm study was the incidence of regorafenib-associated grade 3 HFSR as assessed using the National Cancer Institute (NCI) Common Terminology Criteria for Adverse Events v.4.0. The secondary outcomes were the incidence of all grades of HFSR, time to the development of HFSR of any grade, time to improvement from HSFR **≥** grade 2 to ≤ grade 1, treatment discontinuation rate, treatment interruption rate or dosage reduction due to HFSR, and rate of AEs of aluminum chloride.

### Procedures and follow-up

Follow-up was conducted every week during the first cycle, then every two weeks thereafter for a total of 12 weeks or until treatment discontinuation. The majority of routine, follow-up appointments included a physical examination, laboratory tests (including serum CA19-9 and carcinoembryonic antigen levels), chest radiography, and computed tomography. Dermatological examinations included an assessment of the HFSR symptoms, adverse effects of aluminum chloride ointment, and the presence of other skin lesions. In addition, the palms and soles were photographed every one to two weeks by three dermatologists using a digital camera. HFSR grading was performed in a central review using clinical photographs. If disagreement occurred, the final assessment was decided by the majority (two persons); if all three persons disagreed, a discussion was held until a consensus was reached.

### Statistical analysis

Based on the results of a previous Japanese clinical trial, [10, 11] the threshold value was set at 20% and the expected value was set at 5%. Under these conditions, with α = 0.1 (one-sided) and β = 0.1, the required number of subjects was determined to be 25. After making allowances for ineligible subjects, the enrollment target was increased to 28. For the primary endpoint, an exact binomial test (one-sided, lower-tailed significance level, 10%) assuming a threshold value of 20% was conducted. Point estimates and confidence intervals were estimated for all items of interest, including the primary endpoint. The median duration and confidence interval were estimated using the Kaplan-Meier method and Greenwood’s formula. All statistical analyses were performed using SAS (version 9.4; SAS Institute, Cary, NC, USA).

## Results

### Patient characteristics

In total, 28 patients with a median age of 56.5 years (range: 41.0–81.0 years), including 12 male patients (42.9%), were enrolled between January 2019 and January 2022 (Table 1). The first patient was registered on 08/09/2019. One patient was excluded prior to regorafenib administration owing to disease progression. Table 1 summarizes baseline patient demographic data and clinical characteristics. The vast majority of patients had an Easter Cooperative Oncology Group performance status of 1. Regorafenib was used as a third-line or later treatment. Sixteen patients (57%) had skin lesions before the start of the study treatment and were treated with a topical antifungal agent, topical moisturizer or clavus shaving.

**Table 1:** Baseline patient characteristics

Characteristics	No (%) (n = 28)
Age, median (range), years	56.5 (41.0–81.0)
Sex	
Male	12 (42.9%)
Female	16 (57.1%)
BMI, mean (SD), kg/m^2^	23.1 (4.3)
ECOG performance status	
0	24 (85.7%)
1	4 (14.3%)
Regorafenib therapy	
3rd line	10 (35.7%)
4th line	9 (32.1%)
5th line	7 (25.0%)
≥ 6th line	2 (7.1%)
Skin lesions before study commencement	
None	12 (42.9%)
Present	16 (57.1%)
Tinea	3 (10.7%)
Eczema	5 (17.8%)
Corn/clavus	8 (28.6%)
Other (e.g., hyperkeratosis)	6 (21.4%)

Abbreviations: BMI, body mass index; ECOG, Easter Cooperative Oncology Group; e.g., example; SD, standard deviation

### Regorafenib treatment

Twenty-seven patients received regorafenib, and six patients completed the study during the 12-week observation period. In addition, 22 patients (82%) required treatment interruption, and four patients (15%) required a dosage reduction (see Table 2. Twenty-three (85%), 14 (52%), and 12 (44.4%) patients continued regorafenib without a dosage reduction or treatment interruption in the first cycle through day 7, day 15, and day 21, respectively (Table 2).

**Table 2:** Safety data

Characteristics	No. (%) (n = 27)
Regorafenib AE	
HFRS (All grades)	18 (66.7%)
Grade 1	6 (22.2%)
Grade 2	10 (37.0%)
Grade 3	2 (7.4%)
Hepatic dysfunction	10 (37.0%)
Hypertension	7 (25.9%)
Erythema multiforme	3 (11.1%)
Nephropathy/proteinuria	3 (11.1%)
Thrombocytopenia	2 (7.4%)
Fatigue	2 (7.4%)
Maculopapular drug eruption	1 (3.7%)
Diarrhea	1 (3.7%)
High fever	1 (3.7%)
Nosebleed	1 (3.7%)
Aluminum chloride AE	
Irritation (All grades)	13 (48.1%)
Grade 1	9 (33.3%)
Grade 2	4 (14.8%)
Dry skin (All grades)	16 (59.2%)
Grade 1	15 (55.6%)
Grade 2	1 (3.7%)
Duration of study treatment, median (range), weeks	7.3 (2.1–12.0)
Study treatment	
Complete	6 (22.2%)
Discontinue	21 (77.8%)
Interruption	
Yes	22 (81.4%)
No	5 (18.5%)
Dose reduction	
Yes	4 (14.8%)
No	23 (85.2%)
Patients who continued regorafenib with neither dosage reduction nor interruption during first cycle	
Day 8	23 (85.2%)
Day 15	14 (51.8%)
Day 22	12 (44.4%)

Abbreviations: AE, adverse event; HFSR, hand-foot syndrome

### Clinical outcomes

Among the 27 patients who received treatment, the incidence of grade 3 or higher HFSR, which was the primary endpoint of the present study, was 7.4% (80% confidence interval [CI]: 2.0–18.5%), and the result of the exact binomial test assuming a threshold of 20% was statistically significant (P = 0.0718; Table 2). The median time to the development of HFSR of any grade was 15 days (95% CI: 8.0–47.0 days; Table 3 and eFigure 2 [see Additional file 1]). The median time to improvement from HFSR **≥** grade 2 to ≤ grade 1 was eight days (95% CI: 4.0–10.0 days; Table 2 and eFigure 3 [see Additional file 1]).

**Table 3:** Analysis results

Event			Previous clinical trials
HFSR incidence ratio			
All grades		66.7% (95% CI: 46.0–83.5%)	76%^6^, 58.2%^7^
Grade 1		22.2% (95% CI: 9.6–42.3%)	
Grade 2		37.0% (95% CI: 19.4–57.6%)	
Grade 3		7.4%^*^ (80% CI: 2.0–18.5%)	21%^6,^ 19%^7^
Incidence of regorafenib dosage change (discontinuation/interruption/dosage reduction) due to HFSR			
Discontinuation		0%	
Interruption		11% (95% CI: 2.4–29.2%)	
Dosage reduction		0%	
Incidence of regorafenib dosage changes due to adverse effects related to aluminum chloride		0%	
Time to development of HFSR of any grade, median days		15.0 days (95% CI: 8.0–47.0)	7.0 days [17]
Time to improvement from HFRS **≥** grade 2 to ≤ grade 1, median days		8.0 days (95% CI: 4.0–10.0)	

*: The result of the exact binomial test assuming a threshold of 20% was statistically significant (P = 0.0718)

Abbreviations: HFSR, hand-foot skin reaction; CI, confidence interval; d, day; NC, not calculated.

Table 2 and eFigures 4 and 5 [see Additional file 1] show patient safety data. AEs associated with regorafenib occurred in 22 of 27 (81%) patients, with HFSR of any grade being the main AE in 18 (67%) patients. Grade 1, 2, and 3 HFSR was observed in six, ten, and two patients, respectively. Among AEs other than HFSR, hepatic dysfunction was the most common and was observed in ten patients (36%), followed by hypertension, renal dysfunction, and erythema multiforme in seven (26%), three (11%), and three patients (11%), respectively. Aluminum chloride-related AEs were irritant dermatitis in 13 patients (48%) and dry skin in 16 patients (37%). Grade 2 AEs related to aluminum chloride included irritant dermatitis in four patients (15%) and dry skin in one patient (4%). However, both conditions improved with the administration of strong, topical steroids and moisturizers, which enabled continued use of topical aluminum chloride.

Table 4 shows the reasons for discontinuation, treatment interruption, and dosage reduction. The most common reason for discontinuing regorafenib was disease progression (14 of 21, 67%). Other reasons for discontinuation included hepatic dysfunction (three of 21, 14%) and renal dysfunction, hypertension, and high fever in two patients (7%) each. In terms of skin-related AEs, two patients required treatment discontinuation due to erythema multiforme. No patients required treatment discontinuation due to HFSR.

**Table 4:** Reasons for treatment adjustment

Characteristics			No.
Discontinuation of regorafenib			21
	Reason for discontinuation (includes duplication)		
	Progression of primary disease		14
	Hepatic dysfunction		3
	Hypertension		2
	Nephropathy/proteinuria		2
	High fever		2
	Erythema multiforme		2
	Diarrhea		1
Interruption of regorafenib			22
	Reasons for interruption (includes duplication)		
	Hepatic dysfunction		9
	Hypertension		6
	High fever		6
	Nephropathy/proteinuria		4
	HFSR		3
	Erythema multiforme		2
	Thrombocytopenia		2
	Diarrhea		1
	Fatigue		1
	Nosebleed		1
Dose reduction of regorafenib			4
	Reasons for dose reduction (includes duplication)		
	High fever		2
	Hypertension		2
	Nephropathy/proteinuria		1
	Thrombocytopenia		1
	Fatigue		1

Abbreviations: HFSR, hand-foot syndrome

Treatment interruption occurred in 22 patients (82%) and dosage reduction occurred in four patients (15%; Table 3). The most common reason for treatment interruption was liver dysfunction (nine patients, 41%) while only three patients required treatment interruption due to HFSR (14%). The most common reasons for dosage reduction were high fever and hypertension. No patients required dosage reduction due to HFSR. No patients required treatment discontinuation, interruption, or reduction in the regorafenib dosage due to AEs related to aluminum chloride use.

## Discussion

The present study tested whether topical aluminum chloride, which suppresses sweating in the palms and soles, can prevent the development of regorafenib-related HFSR. The results demonstrated that the incidence of grade 3 HFSR, the study’s primary endpoint, was 7.4%, or well below the 20% threshold of previous clinical trials [6, 7] (P = 0.0718), thereby demonstrating the efficacy of this treatment. However, because regorafenib treatment is often interrupted, reduced, or discontinued, and the median duration of treatment in clinical trials is seven weeks, [3] the assessment of the incidence of grade 3 HFSR over the 12-week observation period in the current study may have limited implications. Indeed, 21 of 27 patients in this study discontinued treatment, and it is unclear whether the incidence of grade 3 HFSR would have remained suppressed or would have increased if these patients had been able to continue treatment for all 12 weeks. Nevertheless, considering that grade 3 HFSR occurred on days 14–21 (weeks 2–3) of the first cycle in all the patients, and that nearly 90% (12 of 14) of the patients in the aforementioned dosage reduction study had grade 3 symptoms during the first cycle of treatment, [6] our results should be valuable to clinicians using regorafenib even if the number of patients continuing treatment for 12 weeks was small.

The regorafenib dosage may be reduced or interrupted or the therapy may be permanently discontinued to manage treatment-related AEs, which typically occur within the first treatment cycle. In a phase III trial of colorectal cancer treatment, the median time to the first occurrence of AEs was 15 days [2]. In the present study, 52% of patients (14 of 27) continued treatment with regorafenib without a dosage reduction or treatment interruption through day 15 of the first cycle, compared to 33% of patients (23 of 70) in previous clinical trials receiving a lower regorafenib dosage of 120 mg [6]. Since the criteria for regorafenib dosage reduction, treatment interruption, and treatment discontinuation used in this study are comparable to those of previous studies, the fact that many patients were able to continue treatment without a dosage reduction or withdrawal in the first 15 days may indicate that aluminum chloride is effective in suppressing the HFSR development.

Previous clinical trials reported the time to HFSR development of any grade as two weeks or less, [4, 5] with a median duration of seven days according to data from clinical trials enrolling Japanese subjects, who are considered to have a high incidence of HFSR [17] (Table 3). In the present study, the median time to the onset of HFSR symptoms was 15 days, which was longer than in the aforementioned clinical study with Japanese subjects [17]. This result may also be attributed to the beneficial effects of aluminum chloride.

The main side effect of topical, aluminum-based, antiperspirant therapy is local skin irritation, which is rarely severe and improves quickly with topical steroids [18]. Application of white Vaseline before applying aluminum chloride is effective in preventing skin irritation [18]. In the present study, dermatitis occurred in approximately half the patients, and grade 2 irritant dermatitis occurred in 14% of the patients. However, the symptoms were mild and improved with topical steroid application. Furthermore, no serious side effects necessitating the discontinuation of aluminum chloride treatment were observed. These results indicate that aluminum chloride ointment can be used relatively safely in patients without hyperhidrosis who have previously received chemotherapy.

The present trial has several limitations, including its small sample size; non-randomized, open-label design; low ethnic diversity; and use of historical data to estimate study endpoints. Other important limitations include the lack of comparable data on the incidence of grade 3 HFSR in patients receiving reduced-dosage regorafenib (120 mg) in phase 3 trials; high frequency of regorafenib discontinuation for reasons other than HFSR; low proportion of patients completing the 12-week follow-up period; and assessment of HFSR using NCI-CTACE, version 4.0 without assessing QOL with tools, such as the hand-foot skin reaction and QOL questionnaire [19].

## Conclusions

The suppression of perspiration with aluminum chloride may prevent progression to grade 3 regorafenib-related HFSR and prolong the time to HFSR development. However, since many subjects in the present study discontinued the treatment during the 12-week study period, further studies enrolling a larger pool of patients receiving regorafenib and other MKIs are required to determine the efficacy of aluminum chloride in reducing the incidence of treatment-related HFSR.

## Electronic supplementary material

Below is the link to the electronic supplementary material.

Additional file 1: **eTable 1** Eligibility criteria **eTable 2** Experimental study **eTable 3** Criteria for treatment interruption, dosage reduction, and discontinuation **eTable 4** Aluminum chloride ointment dispensing method **eFigure 1** Application of dressing materials. Dressing materials were applied to the affected area. **eFigure 2** Time to onset of hand-foot skin reaction **eFigure 3** Time to improvement from hand-foot skin reaction ≥grade 2 to ≤grade 1 **eFigure 4** Clinical appearance of adverse events related to aluminum chloride use A: Grade 2 dry skin; B: Grade 2 irritation **eFigure 5** Clinical appearance of hand-foot skin reaction related to regorafenib use A: Grade 1, B: Grade 2, C: Grade 3

## Data Availability

Data supporting the findings of this study are available from the corresponding author upon reasonable request.

## List of abbreviations

AE: Adverse events
CRC: Colorectal cancer
HFSR: Hand-foot skin reactions
MKI: Multikinase inhibitor
NCI: National Cancer Institute
QOL: Quality of life
UBC: Urea-based topical creams
