# Supplementary material for: Efficacy of aluminum chloride in severe regorafenib-associated hand-foot skin reactions: a single-arm trial
Source: BMC Cancer. 2023 May 4;23:401. doi: 10.1186/s12885-023-10864-9 (PMC10157908; doi:10.1186/s12885-023-10864-9)

**SUPPLEMENTAL DATA**

Additional file 1 (.docx)

eTable 1 | Eligibility criteria

eTable 2 | Experimental study

eTable 3 | Criteria for treatment interruption, dosage reduction, and discontinuation

eTable 4 | Aluminum chloride ointment dispensing method

eFigure 1 | Application of dressing materials

Dressing materials were applied to the affected area.

eFigure 2 | Time to onset of hand-foot skin reaction

eFigure 3 | Time to improvement from hand-foot skin reaction **≥**grade 2 to ≤grade 1

eFigure 4 | Clinical appearance of adverse events related to aluminum chloride use

A: Grade 2 dry skin; B: Grade 2 irritation

eFigure 5 | Clinical appearance of hand-foot skin reaction related to regorafenib use

A: Grade 1, B: Grade 2, C: Grade 3

eTable 1. Eligibility criteria

Inpatients and outpatients with colorectal cancer at the Cancer Institute Hospital who met the following eligibility criteria without meeting any of the exclusion criteria were included.

Inclusion Criteria:

1. Colorectal cancer patients scheduled to receive regorafenib
2. Patients scheduled to receive a regorafenib 120 mg
3. Age 20 to 99 years
4. PS 0–1
5. Provision of written informed consent

Exclusion criteria:

1. Fissures or erosions in the palmoplantar area
2. History of sympathectomy
3. Other conditions determined by the physician-in-charge to be unsuitable for participation in this study

eTable 2. Experimental study

Treatment flow

**After registration**

Urea-based cream and aluminum chloride were applied one week prior to the start of regorafenib treatment.

- Urea-based cream was applied to palms and soles twice daily
- Topical aluminum chloride was applied before sleeping.

**After** **the development of hand-foot syndrome ≥ grade 1**

Dressings were applied to affected areas. Potent topical steroids were used as required with dressings. Urea-based creams or aluminum chloride were applied to areas where dressings were not used.

Criteria for discontinuation

1. Adverse events related to regorafenib use
2. Grade 3 dermatitis caused by aluminum chloride
3. Grade 3 dermatitis caused by coating material
4. Progression of primary disease (colorectal cancer)
5. Discontinuation of treatment at the patient's request

eTable 3. Criteria for treatment interruption, dosage reduction, and discontinuation

HFSR due to Regorafenib

| Grade | Frequency of occurrence | Dosage adjustment |
| --- | --- | --- |
|  | Any number of times | Continue same dosage with supportive care for symptom relief. |
| 2 | 1st | Consider reducing dosage by 40 mg |
|  | If no improvement is observed within 7 days  or at recurrence | Interrupt treatment until reduction to ≤ grade 1. Dosage should be reduced by 40 mg at treatment resumption. |
|  | 3rd | Pause treatment until ≤ grade 1. Dosage should be reduced by 40 mg at treatment resumption. |
|  | 4th | Discontinue regorafenib administration. |
| 3 | 1st | Pause treatment for minimum 7 days until ≤ grade 1. Dosage should be reduced by 40 mg at treatment resumption. |
|  | 2nd | Pause treatment for minimum of 7 days until ≤ grade 1. Dosage should be reduced by 40 mg at treatment resumption. |
|  | 3rd | Discontinue regorafenib administration. |

Abbreviations: HFSR, hand-foot skin reaction

Aluminum chloride

| Grade | Adjustment |
| --- | --- |
| 1 or 2 | Continue topical application of aluminum chloride |
| 3 | Discontinue topical application of aluminum chloride |

eTable 4. Aluminum ointment dispensing method

Chemicals used

- Aluminum (III) chloride 6 hexahydrate 30g
- Hydrophilic cream 70g

Method of administration

1. Aluminum (III) chloride 6 hydrate 30 g was crushed in a mortar.
2. The crushed aluminum (III) chloride hexahydrate was mixed with half the amount of hydrophilic cream to be used (35 g) in a 100 g ointment jar using an automatic axial mixer at 2000 rpm for one minute.
3. After mixing well, the remaining hydrophilic cream (35 g) was added and mixing was performed again for one minute.
4. The mixture was stored at room temperature and later mixed again at 2000 rpm for one minute.

Storage

The mixture was stored below 25°C in a refrigerator during periods of high temperatures to prevent separation. If the mixture separated, it was blended with an ointment spatula before use. The recommended dosage for palms and soles was 30 g once daily for 30 days. The storage period was 3 months.

eFigure 1. Application of dressings


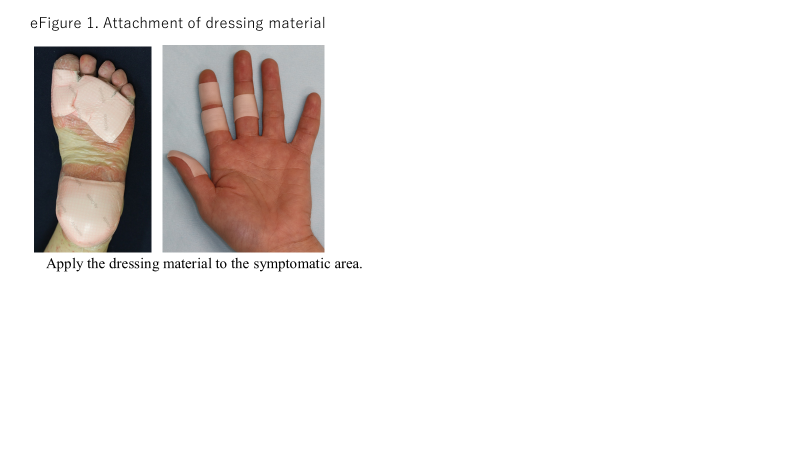


eFigure 2. Time to development of hand-foot skin reactions of any grade
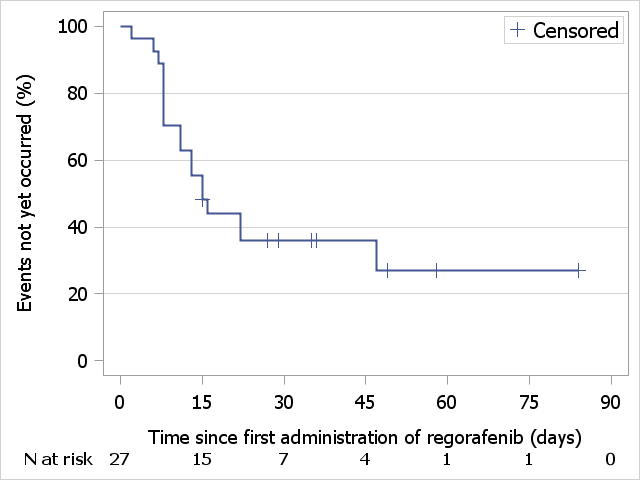


eFigure 3. Time to improvement from hand-foot skin reaction **≥** grade 2 to ≤ grade 1


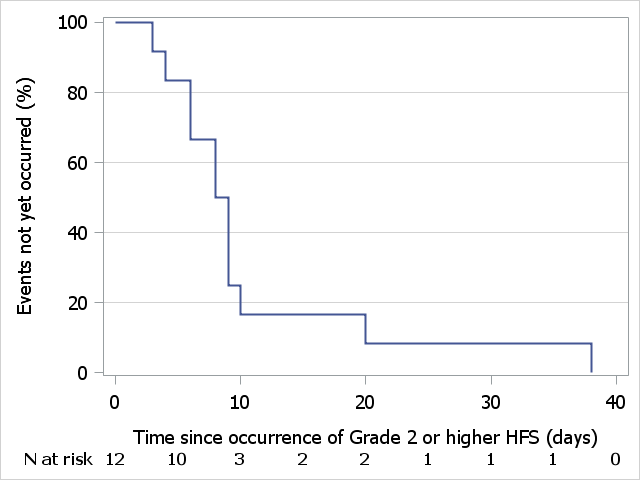


eFigure 4. Clinical appearance of adverse events related to aluminum chloride


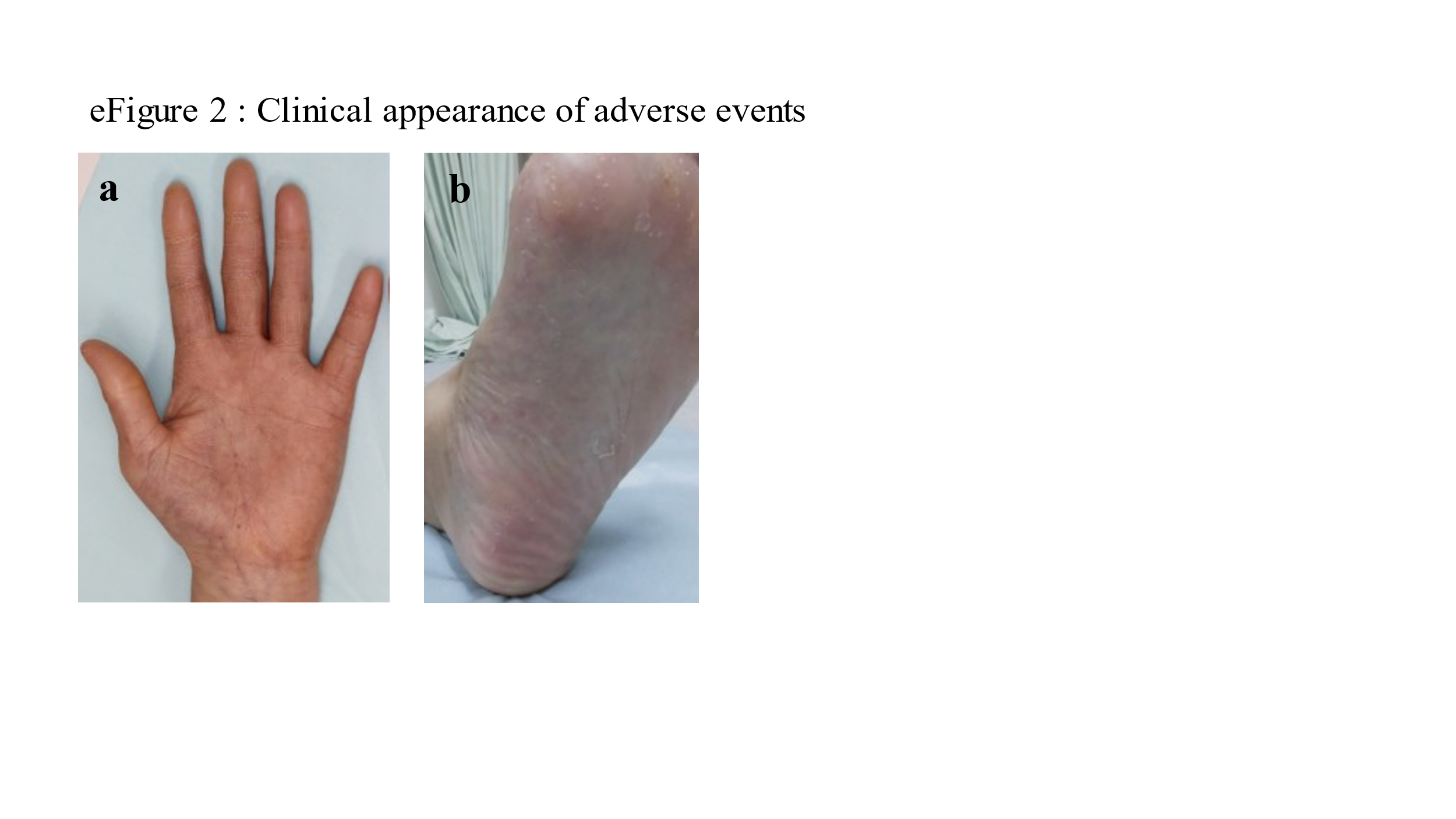


eFigure 5. Clinical appearance of hand-foot skin reactions related to regorafenib use


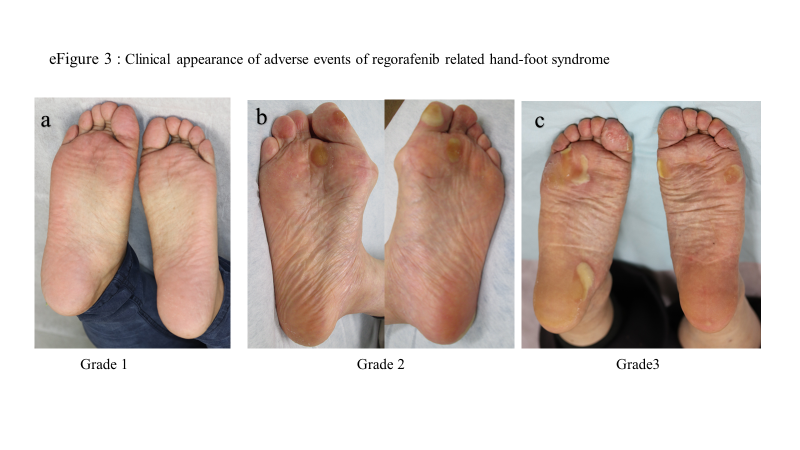

Supplement: Supplementary file 1 — Additional file 1: eTable 1 Eligibility criteria eTable 2 Experimental study eTable 3 Criteria for treatment interruption, dosage reduction, and discontinuation eTable 4 Aluminum chloride ointment dispensing method eFigure 1 Application of dressing materials. Dressing materials were applied to the affected area. eFigure 2 Time to onset of hand-foot skin reaction eFigure 3 Time to improvement from hand-foot skin reaction ≥grade 2 to ≤grade 1 eFigure 4 Clinical appearance of adverse events related to aluminum chloride use A: Grade 2 dry skin; B: Grade 2 irritation eFigure 5 Clinical appearance of hand-foot skin reaction related to regorafenib use A: Grade 1, B: Grade 2, C: Grade 3 [file 12885_2023_10864_MOESM1_ESM.docx]
